# Supplementary material for: Effects of High-Dose Vitamin D Supplementation on Placental Vitamin D Metabolism and Neonatal Vitamin D Status
Source: Nutrients. 2024 Jul 5;16(13):2145. doi: 10.3390/nu16132145 (PMC11243372; doi:10.3390/nu16132145)
Supplement: Supplementary file 1 [file nutrients-16-02145-s001.zip › nutrients-3072893-supplementary.pdf]

Table S1: Clinical characteristics of pregnancies for the 118 placentas studied. Modified from *A technical note to improve the reporting of studies of the human placenta*, Nelson et al [37]. [1]A total of 11 women reported smoking during pregnancy. Four of these were still smoking at time of inclusion (gestational week 11-16), while the remaining seven reported quitting prior to inclusion. [2]At time of inclusion, a total of 44 women reported consumption of alcohol during pregnancy, however the majority (n = 30) only reported an intake prior to knowing about their pregnancy. [3]Reported at time of inclusion. [4]All pregnant women in Denmark are screened for gestational diabetes through urine glucose tests, a minimum of six times during pregnancy. In addition, women with risk factors for gestational diabetes are invited to an oral glucose tolerance test, but we do not have information on whether women included in this study attended this test. However, we do know that none of the women in the present study were diagnosed with gestational diabetes.

**Table 1**				
**Parameter**	**Clinical Characteristics of Pregnancies for Placentas Studied**			
**Gravidity**	Median = 2	25-75% = 1 – 3	Range = 1 – 8	
**Parity**	Median = 1	25-75% = 0 – 1	Range = 0 – 4	
**Gestational age (weeks)**	Average = 40.0	SD = 1.14	Range = 36+1 – 42+1	
**Maternal age (years)**	Average = 30.2	SD = 4.77	Range = 21 – 44	
**Race**	Black, n = 0	White, n = 113	Other, n = 5	
**Ethnicity**	Danish			
**Prenatal medications**	Iron, n = 95	10 ug, Vitamin D = 57<br>90 ug, Vitamin D = 61<br>Calcium, n = 28<br>Multivitamins, n =118<br>Fish oil, n = 34		
**Drugs**	Cigarettes[1],<br>n = 11	Alcohol[2],<br>n = 44	Prescription medicine[3],<br>n = 43	
**Previous prenatal admission(s)**	Yes, n = 6	No, n = 112		
**Blood pressures <140/90 mm Hg**	Yes,n = 118	No, n = 0		
**Screened for diabetes**	Yes[4], n = 118	No, n = 0		
**Antibiotics in labor**	No, n = 95	Penicillin, n = 10	Cefuroxim, n = 13	
**Beta strep status**	Unknown, n = 118			
**Antenatal steroids:**	Yes, n = 0			
**Magnesium sulfate**	Yes, n = 0	No, n = 118		
**Anesthesia**	Epidural, n = 23	Nitrous oxide, n = 45	General, n = 0	Pudendal, n = 2<br>None, n = 52
**Cervical ripening agent**	Prostaglandin E_1,<br>n = 14	Prostaglandin E_2,<br>n = 0	Mechanical<br>n = unknown	
**Labor**	Unknown, n = 118			
**Delivery mode**	C-section = 25	Vaginal = 93		
**Maternal Oxygen given at delivery?**	Unknown			
**Birth weight (grams)**	Average = 3617	SD = 449	Range = 2520 – 4735	
**Placental weight (grams)**	Average = 667.6	SD = 142.3	Range = 370 – 1200	
**Baby's sex**	Female = 65	Male = 53		
**Delivery to processing (mins)**	Average = 136	SD = 64	Range = 20 – 294	

Table S2: Demographic and clinical data for the 118 women with a placenta eligible for gene analysis. Data are presented as mean ± SD or as n (%). Smoking covers women answering yes to smoking at some point during pregnancy and includes both women who were still smoking at time of inclusion and women who had quit prior to inclusion. Non-Scandinavian origin covers women with either one or both parents being born outside Scandinavia (Denmark, Norway, Sweden, or the Faroe Islands). BMI, Body Mass Index.

	10 µg VitD3<br>*n = 57*	90 µg VitD3<br>*n = 61*	*p-value*
Maternal age [years]	29.9 ± 0.56	30.3 ± 0.67	*0.653*
Pre-pregnancy BMI [kg/m^2]	25.8 ± 0.72	26.3 ± 0.81	*0.664*
BMI class [kg/m^2]			*0.995*
Underweight (<18.5)	2 (3.5)	2 (3.3)	
Normal (18.5–24.9)	30 (52.6)	31 (50.8)	
Overweight (25–29.9)	13 (22.8)	14 (23.0)	
Obese (≥ 30)	12 (21.1)	14 (23.0)	
Parity			*0.797*
Nulliparous	22 (38.6)	24 (39.3)	
Primiparous	23 (40.4)	27 (44.3)	
Multiparous	12 (21.1)	10 (16.4)	
Smoking			*0.405*
Yes	4 (7.0)	7 (11.5)	
No	53 (93.0)	54 (88.5)	
Origin of maternal parents			*0.664*
Scandinavian	51 (89.5)	56 (91.8)	
Non-Scandinavian	6 (10.5)	5 (8.2)	
Gestational age at delivery [weeks]	40.2 ± 1.1	39.8 ± 1.2	*0.031*
Mode of delivery			*0.905*
Vaginal	13 (22.2)	12 (19.7)	
Sectio	40 (70.2)	45 (73.8)	
Offspring sex			*0.883*
Boy	26 (45.6)	27 (44.3)	
Girl	31 (54.4)	34 (55.7)	

Table S3: Demographic data for the 101 participants with available first and third trimester samples for VDBP analysis. Data are presented as mean ± SD or as n (%). Seven participants had no information on BMI. Information on age, parity, smoking, origin, and offspring sex was available for all participants. Smoking covers women answering yes to smoking at some point during pregnancy and includes both women who were still smoking at time of inclusion and women who had quit prior to inclusion. Non-Scandinavian origin covers women with either one or both parents being born outside Scandinavia (Denmark, Norway, Sweden, or the Faroe Islands). BMI, Body Mass Index.

	10µg VitD3<br>n=51	90µg VitD3<br>n=50	*p-value*
Maternal age	30.9 ± 3.7	31.0 ± 5.4	0.950
Pre-pregnancy BMI	25.4 ± 5.0	25.6 ± 5.5	0.795
BMI class [kg/m^2]			0.565
Underweight (<18.5)	2 (4.1)	0 (0)	
Normal (18.5–24.9)	28 (57.1)	28 (62.2)	
Overweight (25–29.9)	11 (22.5)	9 (20.0)	
Obese (≥ 30)	8 (16.3)	8 (17.8)	
Parity			0.148
Nulliparous	26 (51.0)	17 (34.0)	
Primiparous	17 (33.3)	26 (52.0)	
Multiparous	8 (15.7)	7 (14.0)	
Smoking			0.538
Yes	6 (11.8)	8 (16.0)	
No	45 (88.2)	42 (84.0)	
Origin of maternal parents			0.007
Scandinavian	50 (98.0)	41 (82.0)	
Non-Scandinavian	1 (2.0)	9 (18.0)	
Offspring sex			0.199
Boy	31 (60.8)	23 (47.9)	
Girl	20 (39.2)	25 (52.1)	

Table S4: Demographic data for the 472 participants with cord blood available for 25(OH)D. Data are presented as mean ± SD or as n (%). Four participants had no information on BMI. Information on age, parity, smoking, origin, and offspring sex was available for all participants. Smoking covers women answering yes to smoking at some point during pregnancy and includes both women who were still smoking at time of inclusion and women who had quit prior to inclusion. Non-Scandinavian origin covers women with either one or both parents being born outside Scandinavia (Denmark, Norway, Sweden, or the Faroe Islands). BMI, Body Mass Index.

	10μg VitD3<br>n=245	90μg VitD3<br>n=227	*p-value*
Maternal age [years]	30.0 ± 4.3	29.9 ± 4.5	*0.862*
Pre-pregnancy BMI [kg/m^2]*	25.6 ± 5.4	26.0 ± 6.1	*0.379*
BMI class [kg/m^2]			*0.584*
Underweight (<18.5)	9 (3.7)	8 (3.6)	
Normal (18.5–24.9)	122 (50.0)	129 (53.1)	
Overweight (25–29.9)	70 (28.7)	52 (23.2)	
Obese (≥ 30)	43 (17.6)	45 (20.1)	
Parity			*0.537*
Nulliparous	117 (47.8)	97 (42.7)	
Primiparous	95 (38.8)	95 (41.9)	
Multiparous	33 (13.5)	35 (15.4)	
Smoking			*0.081*
Yes	19 (7.8)	28 (12.3)	
No	226 (92.2)	199 (87.7)	
Origin of maternal parents			*0.377*
Scandinavian	222 (90.6)	200 (88.1)	
Non-Scandinavian	23 (9.4)	27 (11.9)	
Offspring sex			*0.932*
Boy	121 (49.4)	113 (49.8)	
Girl	124 (50.6)	114 (50.2)	
